# A Chinese White Pear (*Pyrus bretschneideri*) *BZR* Gene *PbBZR1* Act as a Transcriptional Repressor of Lignin Biosynthetic Genes in Fruits

**DOI:** 10.3389/fpls.2020.01087

**Published:** 2020-07-15

**Authors:** Yunpeng Cao, Dandan Meng, Xiaoxu Li, Lihu Wang, Yongping Cai, Lan Jiang

**Affiliations:** ^1^Key Laboratory of Cultivation and Protection for Non-Wood Forest Trees, Ministry of Education, Central South University of Forestry and Technology, Changsha, China; ^2^Central Laboratory, Yijishan Hospital of Wannan Medical College, Wuhu, China; ^3^Key Lab of Non-wood Forest Products of State Forestry Administration, College of Forestry, Central South University of Forestry and Technology, Changsha, China; ^4^School of Life Sciences, Anhui Agricultural University, Hefei, China; ^5^Technology Center, China Tobacco Hunan Industrial Co., Ltd., Changsha, China; ^6^College of Landscape and Ecological Engineering, Hebei University of Engineering, Handan, China

**Keywords:** BZR, expression, microsynteny, duplication, transcriptional repressors

## Abstract

BZR transcription factors play essential roles in plant growth and environmental stimuli, and they are also the positive regulators of Brassinosteroid (BR) signal transduction in diverse plants. In addition, BZR TFs, as crucial regulators of BR synthesis, may have multiple stress-resistance functions and their related regulatory mechanisms have been well illustrated in model plants. Here, we carried out a genome-wide identification of *BZR* members in Chinese pear (*Pyrus bretschneideri*) and identified 13 members. By comparative analysis in five Rosaceae genomes, *BZR* members in the pear genome may have undergone large-scale duplication events during evolution. Purifying selection played an important role in almost all of the orthologous and paralogous gene pairs. According to the expression analysis of the *PbBZRs* during fruit development, three *PbBZRs* were selected for detailed analysis. Transcriptional activation assays presented that *PbBZR1* repressed the promoters of *P. bretschneideri* lignin biosynthetic genes, such as *PbCES9*, *PbCOMT3*, and *PbHCT6*. Our study traces the evolution of *BZR* gene family members in Rosaceae genomes and illustrates that the rates of gene loss and gain are far from equilibrium in different species. At the same time, our results suggest that *PbBZR1* may be involved in the negative regulation of lignin biosynthesis.

## Introduction

Brassinosteroids (BRs) refer to a class of plant-specific steroidal hormones, which play important roles in response to environmental signaling and regulate various growth and developmental processes, including root development, vascular-differentiation, vascular development and senescence, photomorphogenesis, and cell elongation (Clouse et al., 1996; Li and Chory, 1997; Ye et al., 2010; Clouse, 2011). The previously published studies have proven that the BZR protein has a highly conserved N-terminal domain with DNA-binding activity *in vitro* and *in vivo* (He et al., 2005; Yin et al., 2005). The BZR1 DNA binding domain is the most conserved region of the BZR1 proteins and is encoded by the first exon of each gene (He et al., 2005). BZR proteins also contain glycosylation sites of the Glycogen synthase kinase3 (GSK3) family, which consists of 22-24 amino acids. Besides, some BZR members also contain PEST (Proline-Glutamic acid-Serine-Threonine) motifs to control protein stability (Yin et al., 2005). In *Arabidopsis thaliana*, BZR1 and BZR2/BR insensitive 1-EMS-suppressor 1 (BES1) are two widely studied transcription factors in the BR-signaling pathway. There is an atypical basic helix-loop-helix DNA binding motif at the N-terminal of BZR1 and BES1, which can bind to E-box (CANNTG) and BRRE (CGTGT/CG) elements, respectively (He et al., 2005; Yin et al., 2005).

In the BR-signaling pathway, BR binds and activates receptor kinase BR insensitive 1 (BRI1), and the activated BRI1 further interacts with BRI1-associated receptor kinase (BAK1) to activate BR-signaling (Li et al., 2002; Nam and Li, 2002). The activated BRI1 can phosphorylate BR-signaling kinase 1 (BSK1), phosphorylated BSK1 can activate phosphatase BRI1 suppressor 1 (BSU1), and then deactivate BR-sensing protein (BIN2) kinase. Inhibition of BZR1 transcription factor by BIN2 after inactivation disappears, and BZR1 transcription factor was dephosphorylated by Protein phosphatase 2A (PP2A) and enriched in the nucleus (Li et al., 2001; He et al., 2002; Yin et al., 2002; Zhao et al., 2002; Tang et al., 2011). The activated BZR1 and BES1 can bind to specific regions of downstream gene promoters and activate transcription, thereby regulating the expression of BR target genes (Li et al., 2001; He et al., 2002; Yin et al., 2002; Zhao et al., 2002; Tang et al., 2011). In plant BR-signaling pathway, BZR1 and BZR2/BES1 regulate the expression of different traits genes and participate in the regulation of plant growth and development (He et al., 2005; Yin et al., 2005). Jiang et al. (2013) found that the *BZR* gene controlled the size and shape of seeds by regulating the expression of genes such as *MINISEED3* and *HAIKU2*. BZR transcription factors are associated with plant cell growth and photomorphogenesis by interacting with the Phytochrome-interacting factor (*PIF*) family genes and *DELLA* genes (Gallegobartolome et al., 2012; Li et al., 2012; Oh et al., 2012). The heterologous expression of the *A. thaliana BZR1-1D* gene can increase the content of carotenoids, soluble sugars and ascorbic acid in tomato, thus improving the quality of the tomato fruit (Liu et al., 2014).

*BZR* genes were selected for their biological significance. In the present study, taking the *BZR* genes as an example, we studied the evolutionary relationships of these genes in five species, as well as gene duplication and deletion events. It is well known that one or more whole-genome duplication events produced significant fluctuations in the size and arrangement of genomes within the five Rosaceae species (Wu et al., 2013). Our studies trace the differential expansion and retention of the ancestral *BZR* genes in strawberry (*Fragaria vesca*), Chinese plum (*Prunus mume*), peach (*Prunus persica*), European pear (*Pyrus communis*) and Chinese pear (*Pyrus bretschneideri*) and help facilitate the extrapolation of the evolutionary process. Additionally, our data also suggested that PbBZR1 suppressed the expression of the *P. bretschneideri* lignin biosynthetic genes promoters, such as *PbCES9*, *PbCOMT3*, and *PbHCT6*, and provided the basis for the development of a high quality *P. bretschneideri* fruit.

## Materials and Methods

### Sequence Retrieval and Annotation of *BZR* Genes in Five Rosaceae Genomes

The GFF file, cDNA and protein sequences of *P. bretschneideri*, *P. communis*, *P. mume*, *P. persica*, and *F. vesca* were downloaded from GigaDB database (http://gigadb.org/), GDR databse (https://www.rosaceae.org/), PGDD database (http://chibba.agtec.uga.edu/duplication/index/files), Phytozome (https://phytozome.jgi.doe.gov/pz/portal.html), and JGI database (https://jgi.doe.gov/), respectively (Shulaev et al., 2011; Zhang et al., 2012; Verde et al., 2013; Wu et al., 2013; Chagné et al., 2014). The hidden Markov model (PF05687.10) of the BZR domain was downloaded from the Pfam database (http://pfam.sanger.ac.uk/). Then, using PF05687.10 as probe, the proteins database of the five Rosaceae genomes was searched by HMMER 3.0 software (Mistry et al., 2013). Meanwhile, the BZR family protein sequences of *A. thaliana* were used as probes to search the five Rosaceae protein databases by local BLASTP program (E value 10-3).The CDD database (Marchler-Bauer et al., 2014), SMART (Letunic et al., 2012) and Pfam (Punta et al., 2011) were used to confirm the presence of BZR-domain in these identified BZR proteins. The TBtools software was used to visualize gene structure and chromosomes positions of *BZR* genes in these Rosaceae genomes based on the GFF annotation files (Chen et al., 2018a).

### Phylogenetic and Conserved Motif Analysis of BZR Genes in Five Rosaceae Genomes

The BZR protein sequences were subjected to online MEME website (http://meme-suite.org/) with default parameters except that the motif width was set from 6 to 200 and the maximum number of motifs was set to 10 (De Pater et al., 1996). The *P. bretschneideri* BZR protein sequences were aligned with those of *P. communis*, *P. mume*, *P. persica*, and *F. vesca* by MUSCLE software with default parameters (Edgar, 2004). Phylogenetic trees based on the alignment were generated by MEGA 5.0 software with Neighbor-Joining(NJ) method (Tamura et al., 2011), and the Maximum Likelihood (ML) method was also used to construct phylogenetic trees by IQ-tree software (Nguyen et al., 2014) and further to confirm the results from the NJ method. To evaluate the significance of the nodes, we carried out a bootstrap analysis with 1,000 replicates.

### Microsynteny Analysis in Five Rosaceae Genomes

The MicroSyn (Cai et al., 2011) and MCScanX software (Wang et al., 2012) were used to detect the microsynteny within five species. At the beginning of the microsynteny analysis, we prepared three property files (i.e. gene identifier file, gene list file and the CDS file) based on the previously published manuscripts (Cao et al., 2016a; Cao et al., 2016d). By loading these files, the microsynteny diagrams were achieved. In this study, if a region contained three or more conserved homologous gene pairs were located within ten genes upstream and downstream between genomes, then this region is defined as a syntenic block. If gene pairs are derived from microsynteny regions, they may have evolved from a recent common ancestor. To further understand the evolutionary mechanism of the *BZR* genes in the five Rosaceae genomes, we performed genome-wide analysis to determine whether *BZR* genes occurred within syntenic blocks.

### ω and Ks Analysis

As previously published articles (Han et al., 2016), the Ka/Ks values have been widely used to explore the gene evolutionary rate and selection pressure. In general, Ka/Ks < 1 indicates functional constrain with the negative/purifying selection, Ka/Ks > 1 suggests accelerated evolution with the positive selection, and Ka/Ks = 1 indicates the genes are drifting neutrally (Yang, 2007). Pairwise protein sequence was first aligned using MUSCLE software, then the multiple sequence alignments of amino acid and the corresponding CDS sequences were converted to codon alignments using MEGA 5.0 software(Tamura et al., 2011). The DnaSP software was used to calculate the Ka (non-synonymous substitution rate) and Ks (synonymous substitution rate) (Librado and Rozas, 2009). Additionally, we estimate the ω (i.e. Ka/Ks) of the gene pairs for all the orthologs and paralogs to further check whether positive selection acts upon specific sites. In the present study, any Ks values > 2.0 were discarded because of the risk of saturation (Maher et al., 2006).

### Analysis and Data Mining of *PbBZRs* Expression Data From Public Databases

RNA-seq data for different tissues were retrieved from NCBI SRA database. The transcript abundance values obtained for *PbBZR* genes were computed from FPKM values by using TopHat2 and Cufflinks software (Trapnell et al., 2012; Kim et al., 2013). To visualize the gene expression profiles of *PbBZR* genes, the R software was used in the present study.

### RNA Extraction and qRT-PCR Assay

Total RNA from tested tissues was extracted by RNAiso (TaKaRa). According to the manufacturer’s instructions, the first-strand complementary DNA (cDNA) synthesis was carried out with 1 μg total RNA by the PrimeScript™ RT reagent Kit (TaKaRa). The qRT-PCR was performed on the ABI 7500 real-time PCR machine with a total 20 μL reaction, which contained SYBR (TaKaRa) 10 μL, 10 mM forward and reverse primer 0.4 μL respectively, and diluted cDNA 0.1 μL. The *ACTIN* gene was adopted as the internal control for normalization (Cao et al., 2018). The qRT-PCR results were obtained from three biological replicates. The relative expression levels were summaried based on the 2−ΔΔct method (Livak and Schmittgen, 2001).

### Virus Induced Silencing (VIGS)

We used the Primer-BLAST to design gene-specific primers for cloning the coding sequences of *Pbr005006.1* (*PbBZR1*), *Pbr017252.1* (*PbBZR2*), and *Pbr035288.1* (*PbBZR3*) (**Table S4**). Then these target genes were cloned into the pTRV2 vector, respectively. The three recombinant plasmids (pTRV2-*PbBZR1*, pTRV2-*PbBZR2*, and pTRV2-*PbBZR3*), and a control (a pTRV2 empty plasmid) were separately introduced into *Agrobacterium tumefaciens* GV3101 according to the previously published manuscripts (Chen et al., 2018b). Each colony were subsequently incubated overnight at 28°C in 1 mL LB medium having 10 mM MES, 20 mM acetosyringone, 50 mgmL^−1^ rifampicin, 50 mg mL^−1^ gentamicin and 50 mg mL^−1^ kanamycin. VIGS was carried out by infiltration into the fruits of 55-day-old *P. bretschneideri* with Agrobacterium harboring a mixture of pTRV2-target genes and pTRV1 in a 1:1 (v/v) ration.

### Subcellular Localization

The CDS of *PbBZR1*, *PbBZR2*, and *PbBZR3* excluding the stop codon were amplified and together with GFP fragments were cloned into the pCHF3 vector by Infusion (Invitrogen), creating the fusion constructions driven under the 35S promoter (**Table S4**). These recombinant constructs and positive control were subjected to Agrobacterium-mediated transient expression in *Nicotiana benthamiana* leaves. The GFP signals were captured by a Confocal Microscope (TCS-SP8 Leica, Wetzlar, Germany) three days after the infiltrations. The DNA dye 4,6-diamidino-2-phenylindole (DAPI) staining was carried out to indict the nucleus.

### Dual-Luciferase Reporter Assay

For transcriptional activity assay of *Pbr005006.1* (*PbBZR1*), *Pbr017252.1* (*PbBZR2*), and *Pbr035288.1*(*PbBZR3*), a GAL4 binding domain of BD-PbBZR(s) fusion protein was carried out, which can bind to the GAL4 DNA-binding site of the LUC reporter, based on the previously published papers (Chen et al., 2018b) (**Table S4**). To further detect the effects of PbBZR1 on the transcription of*PbHCT6*, *PbCES*9, *PbCCoAMOT1*, *PbCOMT3*, and *PbCCR20*, the*PbBZR1* was fused into pCHF3 vector as effectors. The *PbHCT6*, *PbCES*9, *PbCCoAMOT1*, *PbCOMT3*, and *PbCCR20* promoters were each cloned into pGreen II 0800-LUC vector to create the reporter constructs. According to Sainsbury et al. (2009), all the constructs were transformed into tobacco by Agrobacterium infiltration. The TransDetect Double-Luciferase Reporter Assay Kit (TRANSGEN, China) was used to detect the luciferase activity. Three biological replications per combination were carried out.

### Statistical Analysis

The SPSS 20.0 was used to perform the statistical analyses. The data were analyzed by a two-way ANOVA test. P < 0.05 was considered as the threshold for significance (**P < 0.01 and *P < 0.05).

## Results and Discussion

### Identification of *BZR* Genes in *P. bretschneideri* and Other Four Rosaceae Species

As previously reported, the members of the *BZR* gene family were found to be involved in the regulation of various processes in plants, and the positive roles of the *BZR* genes in BR signal transduction have been well studied in plants, such as *A. thaliana*, *Brassica rapa*, *Eucalyptus grandis* and *Zea mays* (Clouse et al., 1996; Li and Chory, 1997; Ye et al., 2010; Clouse, 2011; Saha et al., 2015; Fan et al., 2018; Manoli et al., 2018; Yu et al., 2018). However, no systematic, in-depth study of the *BZR* gene family has been reported in five Rosaceae species, including *F. vesca*, *P. mume*, *P. persica*, *P. communis*, and *P. bretschneideri*. In our study, six, eight, eight, twelve and thirteen BZR-coding protein sequences were identified in *F.vesca*, *P. mume*, *P. persica*, *P. communis* and *P. bretschneideri*, respectively (**Table S1**). Compared with other gene families in the studied species, the *BZR* gene family is of relatively small size, which is consistent with the previous studies such as six, six, eleven and fifteen *BZR* genes in *A. thaliana*, *E. grandis*, *Z. mays* and *B. rapa*, respectively (Saha et al., 2015; Fan et al., 2018; Manoli et al., 2018; Yu et al., 2018). Interestingly, the numbers of *BZR* genes have a certain degree of expansion in both *P. communis* and *P. bretschneideri*, when compared with those in *F. vesca*, *P. mume* and *P. persica*. The chromosome numbers of *F. vesca*, *P. mume* and *P. persica* were 14, 16 and 16, respectively, while the chromosome number of both *P. communis* and *P. bretschneideri* was 34 (Shulaev et al., 2011; Zhang et al., 2012; Verde et al., 2013; Wu et al., 2013; Chagné et al., 2014), implying that the members of *BZR* gene family might have undergone an expansion corresponding to chromosome number variation. Additionally, we also noted that the pear genomes had undergone the recent whole-genome duplication event but not in *F. vesca*, *P. mume*, and *P. persica*, indicating that this event might contribute to the expansion of *BZR* gene family members in both *P. communis* and *P. bretschneideri*.

To detect the distribution of *BZR* gene family members on chromosomes among *F. vesca*, *P. mume*, *P. persica*, *P. communis*, and *P. bretschneideri*, respectively, we have generated a chromosome map based to the GFF files. In *P. bretschneideri*, two *BZR* gene members were placed on chromosome 10, 11, and 15, and one member on chromosome 2, 3, 5, 6, and 8 respectively, and two gene members located different scaffolds (**Figure S1**). In *P. communis*, two *BZR* gene members were found on chromosome 10 and 15, and one member on chromosome 5, 6, 7, 8, 13, and 17, and remaining gene members located different scaffolds. In *F. vesca*, two *BZR* gene members were distributed on chromosome 3, and one member was found on chromosome 2, 5 and 7, respectively, and one member placed on the scaffold. In *P. mume*, three *BZR* gene members were distributed on chromosome 3, followed by chromosome 5 (two members), and the remaining members were located on chromosome 2, 7, and 8 respectively. In *P. persica*, three *BZR* gene members were found on chromosome 4, and one member placed on chromosome 2, 3, 4, 5, and 7, respectively (**Figure S1**).

### Phylogenetic Analysis of *BZR* Genes in *P. bretschneideri* and Other Four Rosaceae Species

In terms of evolution, *F. vesca*, *P. mume*, *P. persica*, *P. communis*, and *P. bretschneideri* share an ancient whole-genome duplication event (Ks ∼ 1.5–1.8: ∼140 MYA), while the recent whole-genome duplication event is only shared by *P. communis* and *P. bretschneideri* (Ks ∼ 0.15–0.3: 30–45MYA) (Fawcett et al., 2009; Wu et al., 2013). The whole genome duplication events provided conditions for gene duplication, and these duplicated genes have been demonstrated to contribute to functional diversification and innovation during evolution (Cao et al., 2016b; Cao et al., 2017). Duplicated genes and functional diversity lead to more complex organisms. In general, the ancient whole-genome duplication events are considered as a significant source of genomic complexity and functional diversity and are also followed by gene loss events (Bowers et al., 2003; Tang et al., 2008).

**Figure 1:**
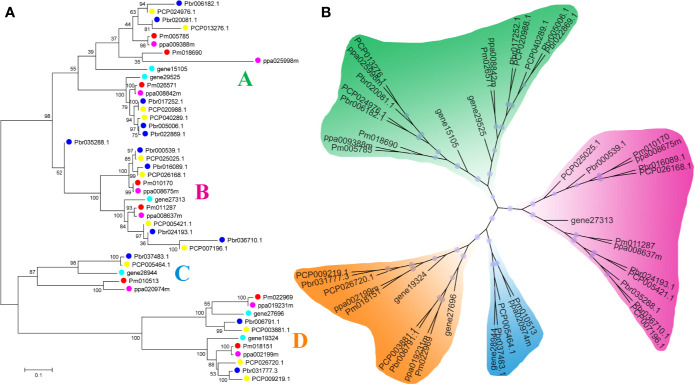
Phylogenetic trees of the five Rosaceae species BZR proteins. **(A)** A phylogenetic tree made by the neighbor-joining method with MEGA 5.0 software. **(B)** A phylogenetic tree generated by the maximum-likelihood method with IQ-tree software. These trees were divided into four groups, designated as A, B, C and D.

**Figure 2:**
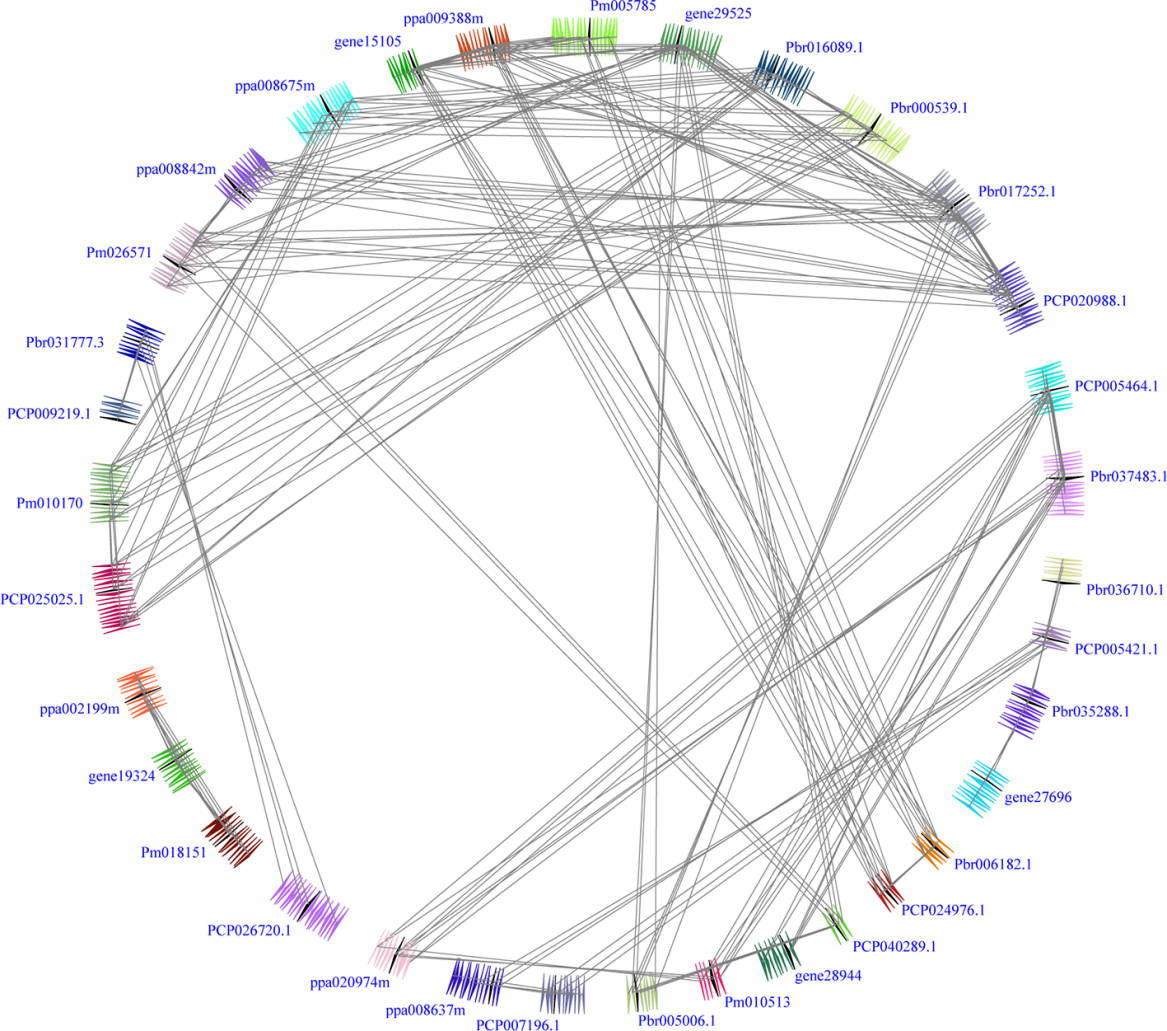
Interspecies microsynteny of *BZR* genes in five Rosaceae genomes, including *Fragaria vesca*, *Prunus mume*, *Prunus persica*, *Pyrus communis* and *Pyrus bretschneideri*. Genomic fragments are suggested by numbers of triangles. Black triangle represented *BZR* genes, and same color indicated these genes in the same fragment.

In our study, two unrooted phylogenetic trees of the *BZR* gene family members were constructed using the Neighbor-Joining (NJ) method with MEGA 5.0, and the Maximum Likelihood (ML) method with IQ-tree, respectively. The tree topologies generated by these two algorithms were similar, except for minor differences in internal branches (**Figure 1**). Therefore, we only consider the NJ tree for further analysis. As shown in **Figure 1**, 47 *BZR* genes could be clustered into four groups (i.e. A-D). Group C contains the fewest *BZR* genes (five), while group A has the most *BZR* genes (seventeen), followed by group B (fourteen) and group D (eleven). Each of these *BZR* genes from the five Rosaceae species contributed at least one *BZR* gene to these four groups, implying that the *BZR* gene family presents a conservative evolutionary trend in Rosaceae species. At the same time, *BZR* gene members from the *P. communis* and *P. bretschneideri* presented higher similarity based on the genetic distance, which was also in keeping with the previously published manuscripts that the closer relationship between *P. bretschneideri* and *P. communis*, versus *P. bretschneideri* and *F. vesca*/*P. mume*/*P. persica*. Additionally, seventeen gene pairs of orthologous genes among the *BZR* gene members, such as *Pbr006182.1* and *PCP024976.1*, and *Pm005785* and *ppa009388m*, while only one paralogous pair (*Pbr005006.1* and *Pbr022869.1*) was identified (**Figure 1**). Subsequently, microsynteny analysis was performed to confirm these results among *F. vesca*, *P. mume*, *P. persica*, *P. communis*, and *P. bretschneideri*. A total of 323 flanking sequences containing *BZR* gene could be assembled into 52 regions (**Figure S2**). *P. bretschneideri* had the most putative duplicated gene pairs, followed by *P. communis* contained four duplicated gene pairs, whereas other three species not contained any duplicated gene pairs (**Figure S2** and **Table S2**). In *P. bretschneideri*, the Ks values of all paralogous pairs varied from 0.1618 to 1.2935 (**Figure 2** and **Table 1**). In *P. communis*, the Ks values of all paralogous pairs varied from 0.1672 to 0.2792. These results suggested that the ancient whole-genome duplication event contributes to the expansion of *BZR* gene family members in both *P. bretschneideri* and *P. communis* (**Figure 2** and **Table 1**). In contrast, the *BZR* gene family members of the other three Rosaceae species have experienced an ancient whole duplication event and followed by gene loss events, which ultimately resulted in fewer members of the *BZR* gene family than *P. communis* and *P. bretschneideri*.

**Table 1:** Duplicated *BZR* gene pairs in *Pyrus bretschneideri* and *Pyrus communis*.

No.	Gene1	Gene2	Ks	Ka	Ka/Ks
1	*Pbr020081.1*	*Pbr006182.1*	0.2691	0.0984	0.3656633
2	*Pbr000539.1*	*Pbr016089.1*	0.218	0.0163	0.0747706
3	*Pbr017252.1*	*Pbr022869.1*	0.1618	0.0171	0.105686
4	*Pbr024193.1*	*Pbr035288.1*	0.4869	0.1213	0.2491271
5	*Pbr024193.1*	*Pbr036710.1*	0.1616	0.0504	0.3118812
6	*Pbr020081.1*	*Pbr022869.1*	1.6293	0.2734	0.1678021
7	*Pbr017252.1*	*Pbr005006.1*	0.1562	0.0142	0.0909091
8	*Pbr006182.1*	*Pbr022869.1*	2.7585	0.3698	0.1340584
9	*Pbr000539.1*	*Pbr036710.1*	1.2935	0.1835	0.1418632
10	*PCP025025.1*	*PCP026168.1*	0.2288	0.0163	0.0712413
11	*PCP024976.1*	*PCP013276.1*	0.2792	0.0709	0.2539398
12	*PCP026720.1*	*PCP009219.1*	0.1758	0.0657	0.3737201
13	*PCP020988.1*	*PCP040289.1*	0.1672	0.0171	0.1022727

**Figure 3:**
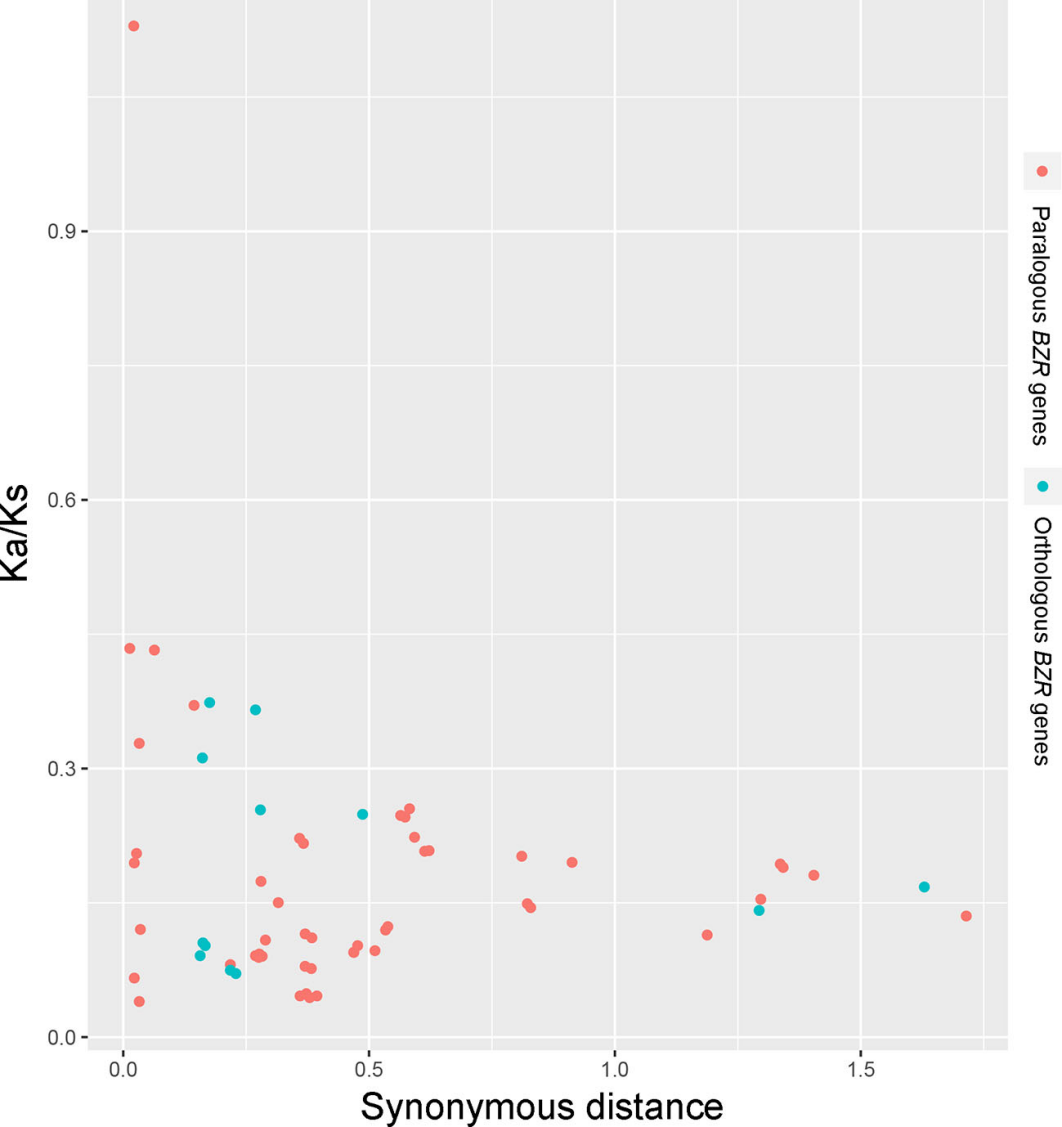
Scatter plots of the Ka/Ks ratios of paralogous *BZR* genes in pear and orthologous *BZR* genes among five Rosaceae genomes. The X-axes and Y-axes indicate the synonymous distance and Ka/Ks ratio for each pair, respectively.

**Figure 4:**
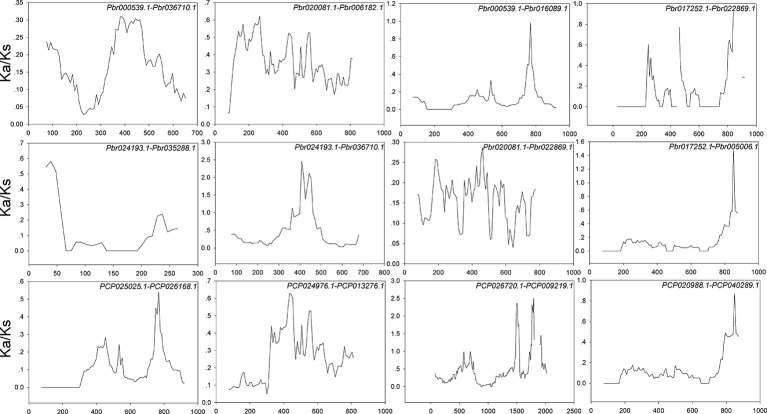
Sliding window plots of duplicated *BZR* genes in *Pyrus bretschneideri* and *Pyrus communis*, respectively. The step size is 9 bp,the window size is 150 bp. The x-axis denotes the synonymous distances within each gene.

Interspecies microsynteny analysis was performed to detect the orthologous *BZR* gene pairs. In total, we identified 34 intraspecies *BZR* genes involved in the interspecies microsynteny (**Figure 3** and **Table S2**). Subsequently, we identified 53 orthologous gene pairs among these five Rosaceae genomes (**Figure 3** and **Table S2**). Among *P. bretschneideri* and other four Rosaceae species (i.e. *F. vesca*, *P. mume*, *P. persica*, and *P. communis*), the different number of orthologous gene pairs were detected, indicating the different loss rates of duplicated genes were occurred during evolution. All paralogous and orthologous gene pairs were detected with Ka/Ks ratios <0.5, except for one orthologous gene pair (*Pbr031777.3*/*PCP009219.1*) with Ka/Ks ratios = 1.1296, clearly suggesting that these gene pairs have undergone strong purifying selection with slowly evolving at the protein level (**Figure 4** and **Table S3**). The sliding window analysis further confirmed that many sites/regions are under neutral to strong purifying selection, as predicted by the Ka/Ks analysis described above (**Figure 4**). Based on this analysis, we found that the majority of Ka/Ks ratios across coding regions were far less than one, except from several distinct peaks (i.e. Ka/Ks >1). These results suggested that positive selection (i.e. Ka/Ks >1) might contribute to a higher Ka/Ks ratio, but will not produce results with a gene average Ka/Ks ratio greater than one. To sum up, evolutionary constraints involved in the evolution of the *BZR* gene family might contribute to the conservation and stability of function in these family members.

### Structural Features of *BZR* Genes in *P. bretschneideri* and Other Four Rosaceae Species

Gene structural diversity is one of the mechanisms for the evolution of multigene families (Cao et al., 2016b; Cao et al., 2017). The exon–intron organization of each *BZR* gene member was analyzed in five Rosaceae species. As shown in **Figure S3**, most of the genes exhibited similar splicing patterns, such 63.8% (30/47) *BZR* genes contained one or two introns. Remarkably, we also found that the intron–exon structure of the sister gene pairs (i.e. orthologous or paralogous genes pairs) was conserved, except for several gene pairs had some differences. For example, *Pm018690* has one intron, while is orthologous gene *ppa025998m* contains three introns, and *Pbr000539.1* has one intron, while is orthologous gene *PCP025025.1* contains two introns. These results implied that both exon loss and gain has occurred during the long evolutionary period of the *BZR* gene family, which further may explain the functional differences of closely related-*BZR* gene family members.

In addition to the exon–intron structure analysis of *BZR* genes, other conserved domains or motifs analyses might also be important for the diversity of BZR protein sequences from *F. vesca*, *P. mume*, *P. persica*, *P. communis*, and *P. bretschneideri*. Next, all the 47 BZR proteins from these five species were subjected to the online MEME website to detect conserved motifs shared among related proteins (**Figure S3**). The conserved motif and multiple sequence alignments analyses showed that most of the BZR proteins contain a typical bHLH domain (basic helix–loop–helix) in these sequences N-termini represented by motif 1 (**Figure S4**). In addition, some BZR proteins contained PEST domain and serine (S)-rich phosphorylation sites, which were represented by the motif 5 and motif 6, respectively, in the middle portions of these sequences (**Figure S4**). The previously published articles showed that PEST domains are contributed to controlling protein stability (Yin et al., 2005). Motif 2 and 4 were found to encode the C-terminal domain, and this region was highly conserved among all of the BZR proteins by multiple sequence alignments. To sum up, the structural analysis of *BZR* genes supported the above result from phylogenetic analysis.

### Expression of *PbBZRs* in Different Tissues

The expression patterns of *BZR* genes in plant organ growth and development have been studied in several species, including *Z. mays*, *E.grandis*, *B.rapa*, and legume (Saha et al., 2015; Fan et al., 2018; Manoli et al., 2018; Yu et al., 2018). To further understand the putative function of all *PbBZR* genes, we examined their expression patterns in four tested tissues, including roots, stems, leaves and fruits in *P. bretschneideri*. The data showed that the expression of different *PbBZRs* varied between roots, stems, leaves and fruits, and some of them were preferentially expressed in specific tissues (**Figure S5**). The previously published manuscripts have confirmed that the *BZR* genes could regulate plant architecture, development of fruit, spikelet and root, and promote cell elongation (Clouse et al., 1996; Li and Chory, 1997; Ye et al., 2010; Clouse, 2011). For example, Lu et al. (2017) found that *GmBZR1* can enhance the size and weight of soybean and ultimately increase its yield (Lu et al., 2017). To further investigate the expression of these *PbBZRs* in *P. bretschneideri* fruit, we detected the relative transcript accumulation of *PbBZRs* at seven stages of development (from very young to mature fruits). Except for *Pbr037483.1*, *Pbr006182.1*, and *Pbr022869.1*, the remaining *PbBZRs* had distinct expression patterns during pear fruit development (**Figure S5**). Remarkably, the expression of *Pbr017252.1*, *Pbr020081.1*, and *Pbr036710.1* was highly expressed during all pear fruit stages of development, indicating that these three genes might play regulatory roles during fruit development in *P. bretschneideri*, such as regulate the cell elongation and senescence. The qRT-PCR was carried out to verify the expression as shown in **Figure S6**. Our data presented that these *PbBZR* genes contained similar expression patterns [Pearson correlation coefficient (PCC) > 0.7] as RNA-seq (**Figure S6**).

Weighted Gene Co-Expression Network Analysis (WGCNA) showed that the *PbBZRs* are in the same module as the key genes of the lignin metabolism pathway, such as *PbCCoAMOT* and *PbCCR*, indicating that they may be involved in regulating lignin biosynthesis (Zhang et al., 2016; Cao et al., 2019). The expression of *Pbr005006.1*(*PbBZR1*), *Pbr017252.1*(*PbBZR2*), and *Pbr035288.1*(*PbBZR3*) exhibited increased accumulation during pear fruit development middle stages (i.e. 55-115 days after flowering), which was basically consistent with our previous findings that the lignin contents of the pear fruit increased from the early to middle stages and decreased during the mature stage (Jin et al., 2013; Cao et al., 2016c). Additionally, Rao and Dixon (2017) revealed that BZRs may be key transcription factors regulating key enzyme genes in the lignin biosynthetic pathway (Rao and Dixon, 2017). Therefore, it is interesting to investigate whether *PbBZRs* can directly bind to the promoters of lignin biosynthesis genes.

Here, we demonstrated that *PbBZRs* were involved in *P. bretschneideri* fruit development, among which the expression of *PbBZR1*, *PbBZR2* and *PbBZR3* presented good correlation with lignin contents changes (Jin et al., 2013), thus these three genes were selected as candidate genes for studying its role in fruit lignin metabolism of *P. bretschneideri*.

### Subcellular Localization and Transcriptional Activity of *PbBZR* Genes

**Figure 5:**
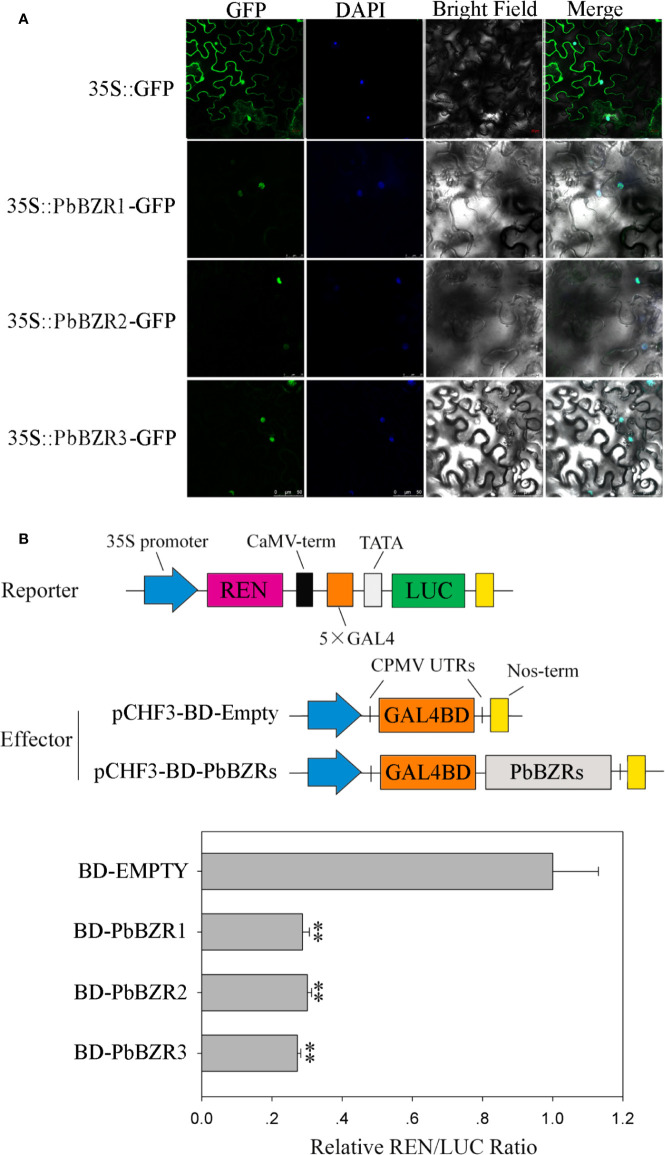
Subcellular localization **(A)** and transcriptional activity **(B)** assay. The green fluorescent protein (GFP) and fusion constructs were driven by the CaMV-35S promoter and were transiently expressed in tobacco leaves. DAPI (dye 4,6-diamidino-2-phenylindole) staining was performed to indicate the nucleus. The transcription repressing activities were indicated as the relative ratio of LUC/REN. Each value is the mean ± SE of three biological replicates. The ** represents significant differences at 0.01 level.

It is reported that BZR proteins were located in nucleus, such as ten maize *BZR* proteins (ZmBZR1, -2, -3, -4, -5, -7, -8, -9, 10 and -11) were targeted to the nucleus (Yu et al., 2018). To determine the subcellular localization of three selected *PbBZR* genes (*PbBZR1*, *PbBZR2* and *PbBZR3*), we constructed three fusion proteins (i.e. 35S:PbBZR1-GFP, 35S:PbBZR2-GFP and 35S:PbBZR3-GFP) for transient transformation *via* Agrobacterium injection methodology. The fluorescence signal of 35S:GFP was found to distribute throughout the cell, whereas the fluorescence signals of the GFP fusion proteins were specifically found to be localized in the nucleus (**Figure 5A**), which was confirmed by DAPI staining. These data suggested that PbBZR1, PbBZR2 and PbBZR3 were nuclear proteins, which were similar to ZmBZRs(Yu et al., 2018). The transcriptional activities of PbBZRs were also tested. A dual luciferase reporter assay was used in plant cells. These data presented that co-expression of these three pBD-PbBZR proteins are able to significantly inhibit the expression of the LUC reporter by co-expression with the individual reporter plasmid, indicating that these BZR transcription factors were possibly transcriptional repressors and had no transactivation activity (**Figure 5B**), which was similar to MaBZR1 and MaBZR2 (Guo et al., 2018).

### Silencing of PbBZR1/2/3 in *P. bretschneideri* Fruit Confers Increased Lignin Contents

**Figure 6:**
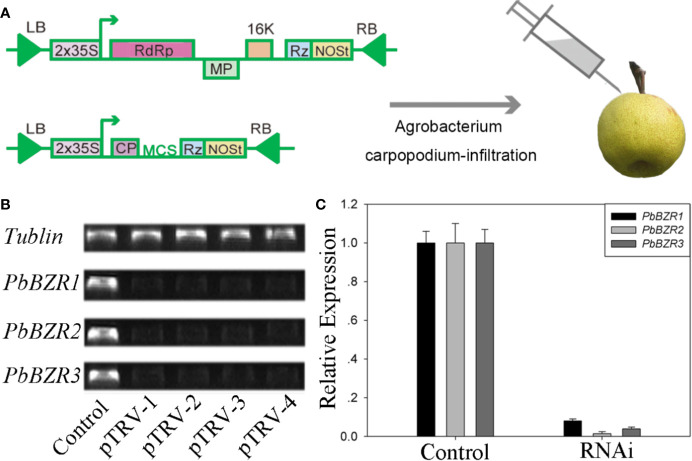
VIGS technique applied to the *Pyrus bretschneideri* fruits. **(A)** Diagram of the VIGS technique used for infecting the *P. bretschneideri* fruits. **(B, C)** The silencing efficiency of the *PbBZR1-3* using semi-quantitative real time PCR (sRT-PCR) and quantitative real time PCR (qRT-PCR). The *ACTIN* gene was adopted as the internal control for normalization (Cao et al., 2018).

**Figure 7:**
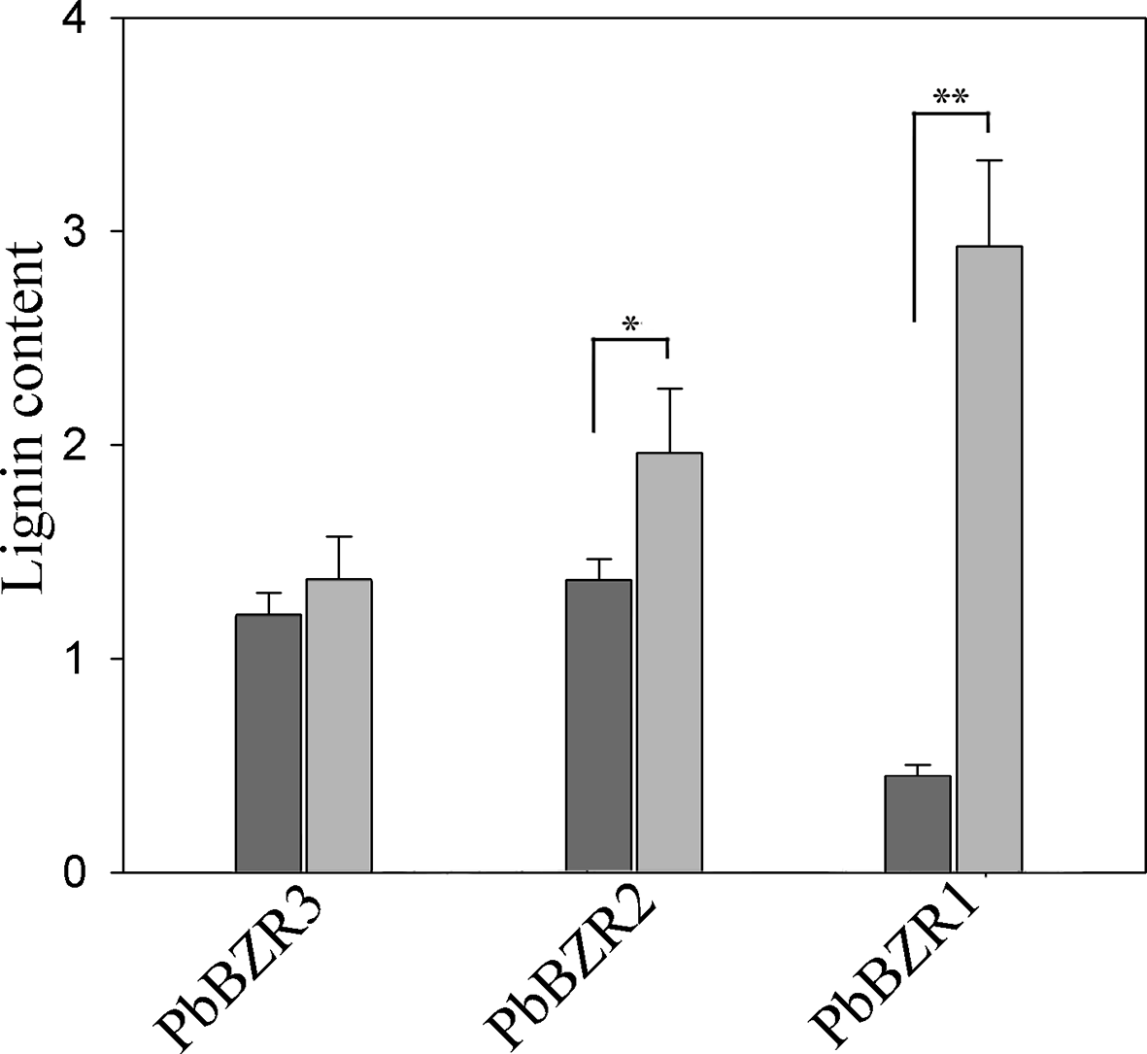
Determination of lignin content. Values are presented as mean ± SE of three biological replicates. Y-axis indicates the relative abundance of *PbBZRs* in leaves of TRV control (black) and TRV-PbBZRs-infected (grey) fruits of 55-day-old after infiltration. The * and ** represent significant differences at 0.05 and 0.01 levels, respectively.

*P. bretschneideri* is a commercial fruit available worldwide. To further understand the role of *PbBZR1/2/3*, we used a VIGS (virus-induced gene silencing) to suppress the expression of *PbBZR1/2/3* in *P. Bretschneideri* fruit (**Figure 6A**). In our study, the 55-days fruits were selected because the lignin content of the *P. bretschneideri* fruit peaked during this period. Subsequently, transcript analysis of the fruits suggested that the expressions for these three genes (*PbBZR1/2/3*) were significantly suppressed in RNAi fruit (**Figure 6**). Given that the silencing of *PbBZR1* led to a significant increase in the lignin content of *P. bretschneideri* fruit (**Figure 7**), the qRT-PCR was used to detect the expression of the related genes in *PbBZR1* silence fruits. Expression of fifteen lignin synthetic genes (*PbPAL2*, *PbC4H1*, *PbC4H3*,*Pb4CL12*,*PbHCT6*, *PbC3H1*, *PbCES*9, *PbCCoAMOT1*, *PbCCoAOMT2*, *PbF5H1*, *PbF5H3*, *PbCOMT3*, *PbCCR20*, *PbCAD1*, and *PbCAD2*) was increased in *PbBZR1-*RNAi fruits (**Figure S7**), which was consistent with the increase of lignin content in the fruits of silencing (Cao et al., 2019). These results indicated that *PbBZR1* might negatively regulate lignin biosynthesis by suppressing these lignin synthetic genes in *P. bretschneideri* fruits, which was in line with the previously published manuscripts (Cao et al., 2019). For example, the *AtBZR1* and its homologous gene *AtBZR2* (*AtBES1*) have been confirmed to directly bind to promoter regions of a large number of lignin-related genes (Jiang et al., 2015), such as *CES* genes (Xie et al., 2011), *MYB* and *NAC* genes (Zhao and Dixon, 2011; Benatti et al., 2012)associated with regulatory pathways for lignin synthesis.

### PbBZR1 Bound to and Suppressed the Promoters of Lignin Biosynthetic Genes

**Figure 8:**
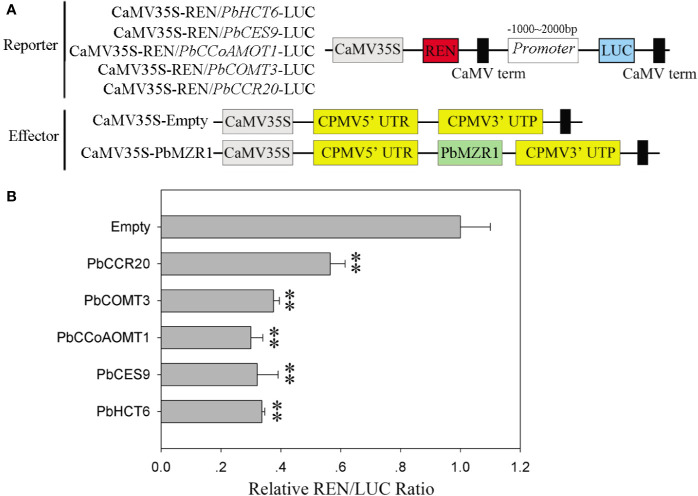
PbBZR1 inhibits the transcription of *PbHCT6*, *PbCES9*, *PbCCoAOMT1* and *PbCCR20*. **(A)** Schematic view of the reporter and effector constructs for the dual-luciferase reporter assay. **(B)** Inhibition of *PbHCT6*, *PbCES9*, *PbCCoAOMT1* and *PbCCR20* expressions by PbBZR1. The inhibition was indicated by the ratio of LUC/REN. The LUC/REN ratio of the empty vector plus promoter was used as the calibrator (set as 1). Values are presented as mean ± SE of three biological replicates. The ** represent significant differences at 0.01 level.

Silencing of *PbBZR1* promotes the expression of lignin synthesis-related genes in RNAi fruits, we used transcriptional activation in *Nicotiana benthamiana* leaves to investigate whether their *P. bretschneideri* counterparts’ promoters could be repressed by *PbBZR1*. The *PbHCT6*, *PbCES*9, *PbCCoAMOT1*, *PbCOMT3*, and *PbCCR20* promoters were cloned and amplified from *P. bretschneideri* gDNAs (**Figure 8A**). These genes were selected, because they had relatively higher expression than other genes in *PbBZR1* mediated RNAi fruits. Interaction of PbBZR1 protein with promoters of *PbHCT6*, *PbCES*9, *PbCCoAMOT1*, *PbCOMT3*, and *PbCCR20* was further confirmed by a transient transcription assay. The promoters of *PbHCT6*, *PbCES*9, *PbCCoAMOT1*, *PbCOMT3*, and *PbCCR20* were individually fused to the LUC reporter gene, to generate the reporters. The effector was PbBZR1, which was driven by the 35S promoter (**Figure 7B**). Each promoter-LUC construct was then co-transferred with 35::PbBZR1, in tobacco leaves. Results showed that PbBZR1 repressed the transcription of LUC reporter gene driven by *PbHCT6*, *PbCES*9, *PbCCoAMOT1*, *PbCOMT3*, and *PbCCR20* promoter (**Figure 8B**). These data suggest that PbBZR1 is transcriptional repressor of *PbHCT6*, *PbCES*9, *PbCCoAMOT1*, *PbCOMT3*, and *PbCCR20* involved in the direct and indirect regulation of lignin biosynthesis during *P. bretschneideri* fruit development. Previous studies have shown that stone cell content is a key factor affecting *P. bretschneideri* fruit flavor (Jin et al., 2013; Cao et al., 2016c). Lignin, a major component of *P. bretschneideri* stone cells, hinders the value and quality of commercial fruit (Jin et al., 2013). Collectively, our research reveals that a regulatory network mediated by *PbBZRs* assists a solid foundation for future functional studies and molecular breeding to improve *P. bretschneideri* fruit quality.

## Conclusions

In our study, a systematic study was performed to identify and characterize the *BZR* genes in five Rosaceae species. The newly identified *BZR* family genes were well studied through phylogenetic analysis, gene structure, microsynteny analysis, selection pressure, expression profiling and subcellular localization analysis, which provided further insights into this gene family. Remarkably, three *PbBZR* genes, including *PbBZR1*, *PbBZR2* and *PbBZR3*, were localized in the nucleus and might be involved in the negative regulation of lignin biosynthesis. Our data mark another step toward the dissection of the molecular network which regulates lignin formation for genetic modification of *P. bretschneideri*.

## Data Availability Statement

P. bretschneideri fruit developmental stage 1 (15DAF), Accession: SRX1595645; P. bretschneiderifruit developmental stage 2 (30DAF), Accession: SRX1595646; P. bretschneiderifruit developmental stage 3 (55DAF), Accession: SRX1595647; P. bretschneideri fruit developmental stage 4 (85 DAF), Accession: SRX1595648; P. bretschneiderifruit developmental stage 5 (115 DAF), Accession: SRX1595650; P. bretschneiderifruit developmental stage 6 (130 DAF), Accession: SRX1595651; P. bretschneiderifruit developmental stage 7 (145 DAF), Accession: SRX1595652; P. bretschneideri leaves_1, Accession: SRR5866154; P.bretschneiderileaves_2, Accession: SRR5866157; P. bretschneideri leaves_3, Accession : SRR5866160; P. bretschneideri roots_1, Accession: SRR5866155; P. bretschneideri roots_2,Accession: SRR5866158; P. bretschneideri roots_2, Accession: SRR5866161; P. bretschneideristem_1, Accession: SRR5866156; P. bretschneideri stem_2, Accession: SRR5866159; P.bretschneideri stem_3, Accession: SRR5866162; P. bretschneiderifruit_1, Accession : SRR4492447; P. bretschneiderifruit_2, Accession: SRR4492450.The authors affirm that all data necessary for confirming the conclusions of the article are present within the article, figures, tables and **Supplementary Materials**.

## Author Contributions

YuC, LJ, LW, and YoC designed and performed the experiments. YuC, LJ, LW, DM, and XL analyzed the data. LJ, LW, YuC and YoC contributed reagents/materials/analysis tools. YuC wrote the paper. All authors contributed to the article and approved the submitted version.

## Conflict of Interest

Author Xiaoxu Li was employed by company China Tobacco Hunan Industrial Co., Ltd. The remaining authors declare that the research was conducted in the absence of any commercial or financial relationships that could be construed as a potential conflict of interest.

## Supplementary Material

Figure S1 Chromosomal location of *BZR* genes. The distribution of *BZR* genes among the chromosomes in each species is diverse. The chromosome number is indicated at the top of each chromosome.

Figure S2 Exon-intron structure and distribution of conserved motifs of *BZR* genes. Left panel: an unrooted phylogenetic tree constructed using MEGA by the N-J method. Clades of *BZR* genes (1–4) are highlighted with different colored backgrounds. Right panel: distribution of conserved motifs and exon-intron structure.

Figure S3 Alignment of deduced amino acid sequences of BZR proteins. (a) represent the basic helix-loop-helix-like structure, (b) indicate serine (S)-rich phosphorylation sites and (c) suggest ‘PEST’ domain.

Figure S4 Figure S4 Intraspecies microsynteny of *BZR* gene families in *P. bretschneideri* and *P. communis*. Genomic fragments are suggested by numbers of triangles. Black triangle represented *BZR* genes, and same color indicated these genes in the same fragment.

Figure S5 Figure S5 The expression of *PbBZRs* in different tissues (a) and fruit development stages (b). The spectrum from blue to red indicates the relative expression level from 0 to 6. Fruit_stage1, Fruit_stage2, Fruit_stage3, Fruit_stage4, Fruit_stage5, Fruit_stage6 and Fruit_stage7 indicated 15 days after flower (DAF), 30 DAF, 55 DAF, 85 DAF, 115 DAF, 130 DAF and145 DAF, respectively.

Figure S6 Figure S6 qRT-PCR verification of 10 pear genes in seven developmental stages of pear fruits, including 15 days after flower (DAF), 30DAF, 55DAF, 85DAF, 115DAF,130DAF and 145 DAF.

Figure S7 Figure S7 qRT-PCR assay of key genes in *P. bretschneideri* lignin metabolic pathway. The Y-axis indicates the relative expression levels. The expression of each gene in control fruits was set as 1. Each value is the mean ± SE of three biological replicates.

Table S1 The detailed information of *BZR* family members in five Rosaceae genomes. The gene IDs of *P. bretschneideri*, *P. communis*, *P. mume*, *P. persica* and *F. vesca* are found in the GigaDB, GDR, PGDD, Phytozome, and JGI, respectively.

Table S2 The large scale duplication events of *BZR* family five Rosaceae genomes. A large scale duplication event was defined as a region where three or more conserved homologs were located within ten genes upstream and downstream between genomes.

Table S3 Ka/Ks analysis of *BZR* gene pairs in five Rosaceae genomes. The DnaSP software was used to calculate the Ka, Ks, and Ka/Ks.

Table S4 Primers in this study. The Primer-BLAST was used to design gene-specific primers for cloning and qRT-PCR.
