# Supplementary figures and images for: A Chinese White Pear (Pyrus bretschneideri) BZR Gene PbBZR1 Act as a Transcriptional Repressor of Lignin Biosynthetic Genes in Fruits
Source: Front Plant Sci. 2020 Jul 15;11:1087. doi: 10.3389/fpls.2020.01087 (PMC7379032; doi:10.3389/fpls.2020.01087)

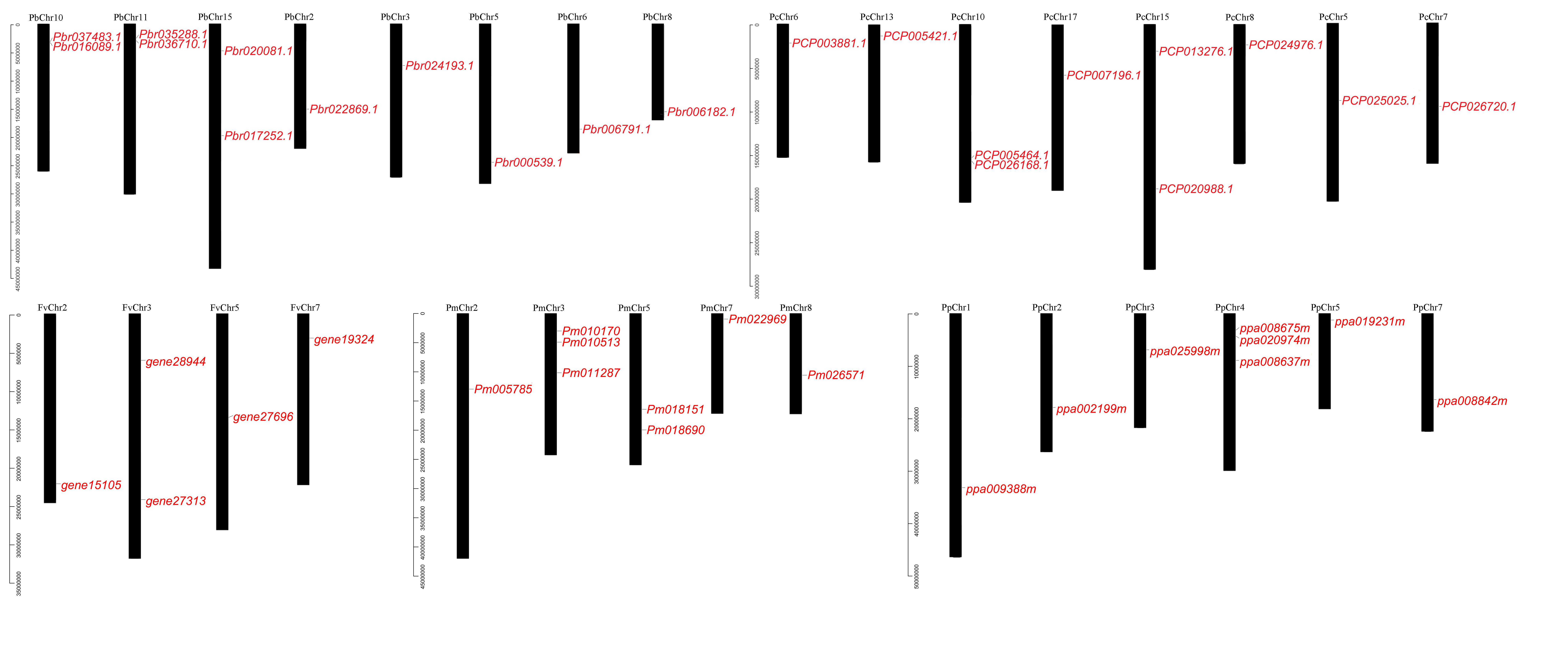

Supplement: Figure S1 — Chromosomal location of BZR genes. The distribution of BZR genes among the chromosomes in each species is diverse. The chromosome number is indicated at the top of each chromosome. [file Image_1.tif]

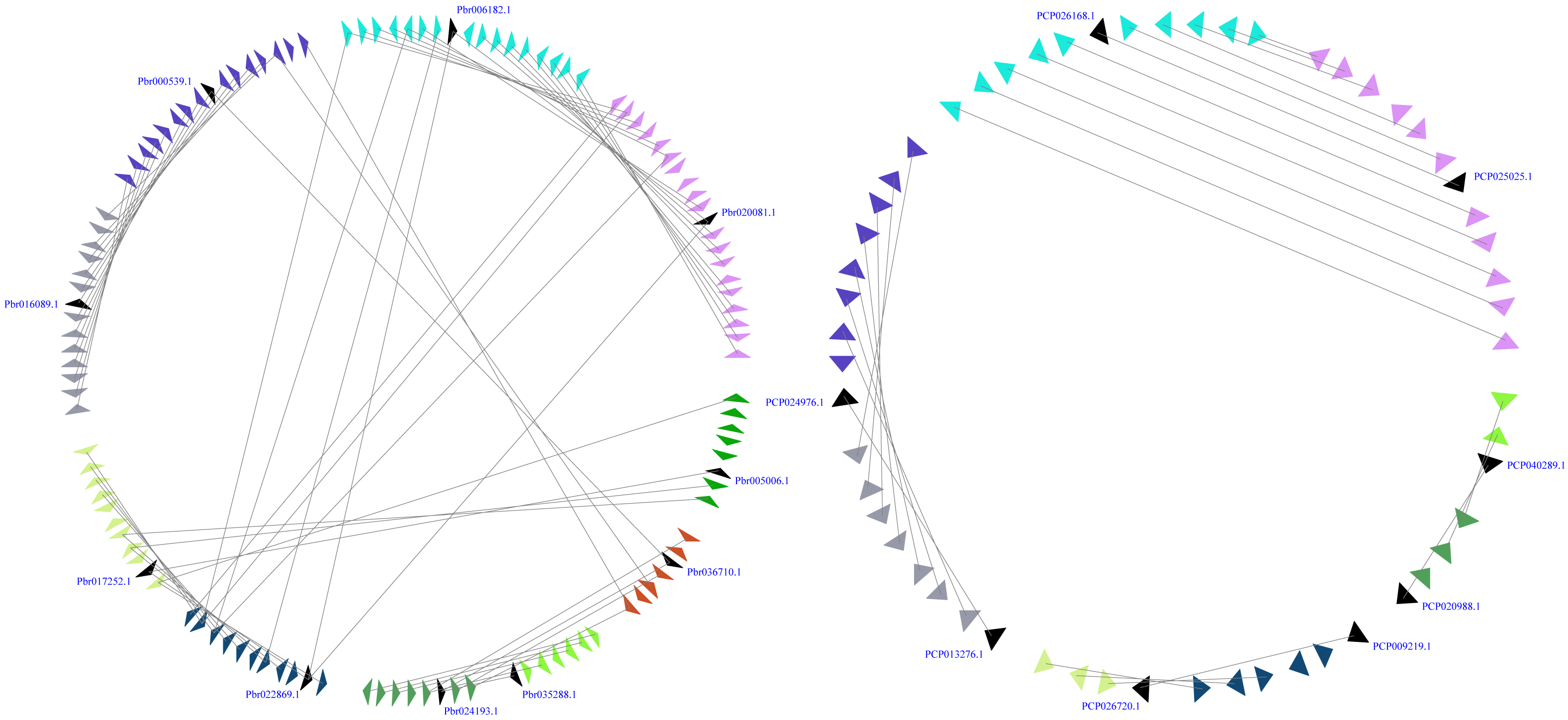

Supplement: Figure S2 — Exon-intron structure and distribution of conserved motifs of BZR genes. Left panel: an unrooted phylogenetic tree constructed using MEGA by the N-J method. Clades of BZR genes (1–4) are highlighted with different colored backgrounds. Right panel: distribution of conserved motifs and exon-intron structure. [file Image_2.tif]

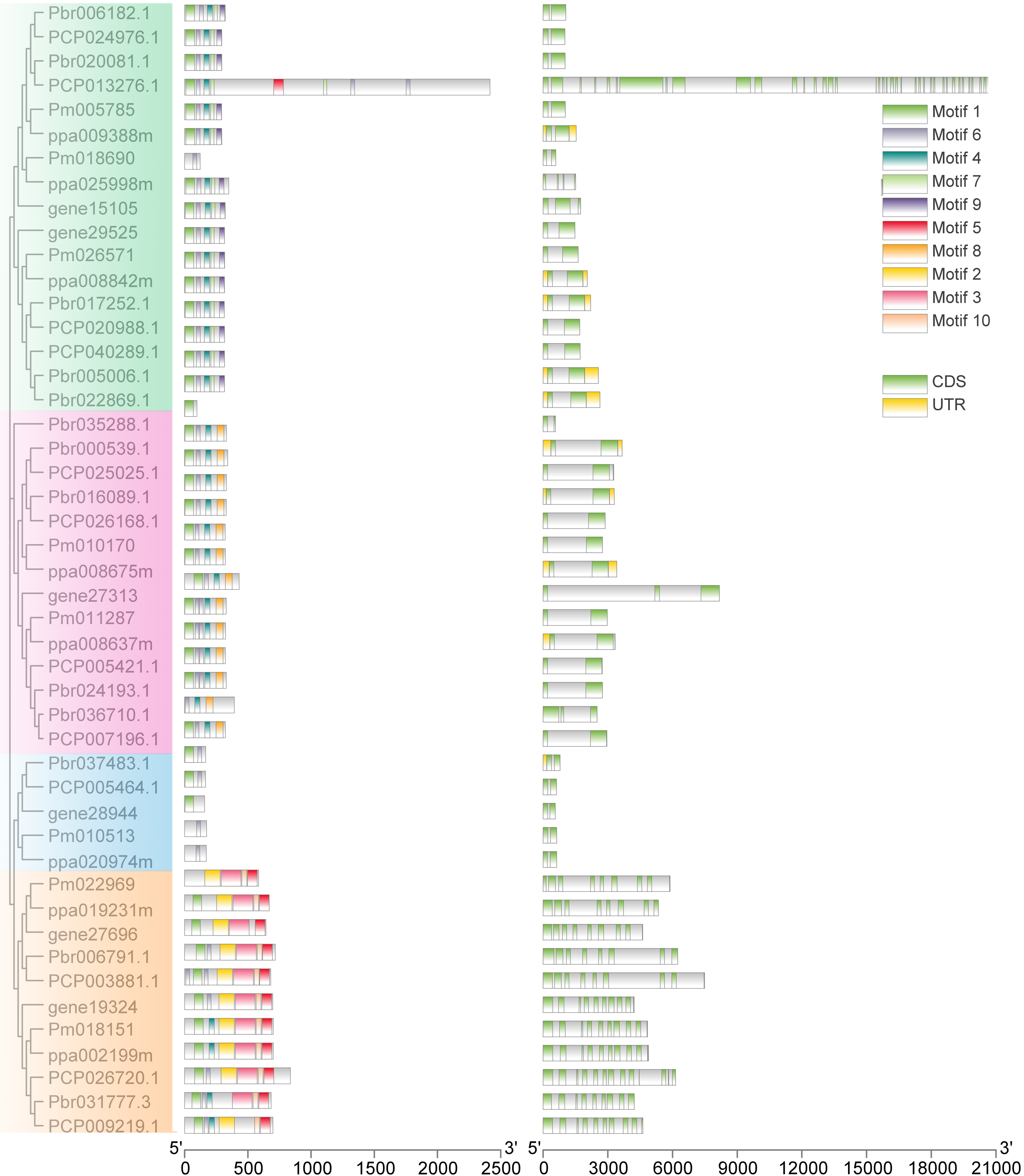

Supplement: Figure S3 — Alignment of deduced amino acid sequences of BZR proteins. (a) represent the basic helix-loop-helix-like structure, (b) indicate serine (S)-rich phosphorylation sites and (c) suggest ‘PEST’ domain. [file Image_3.tif]

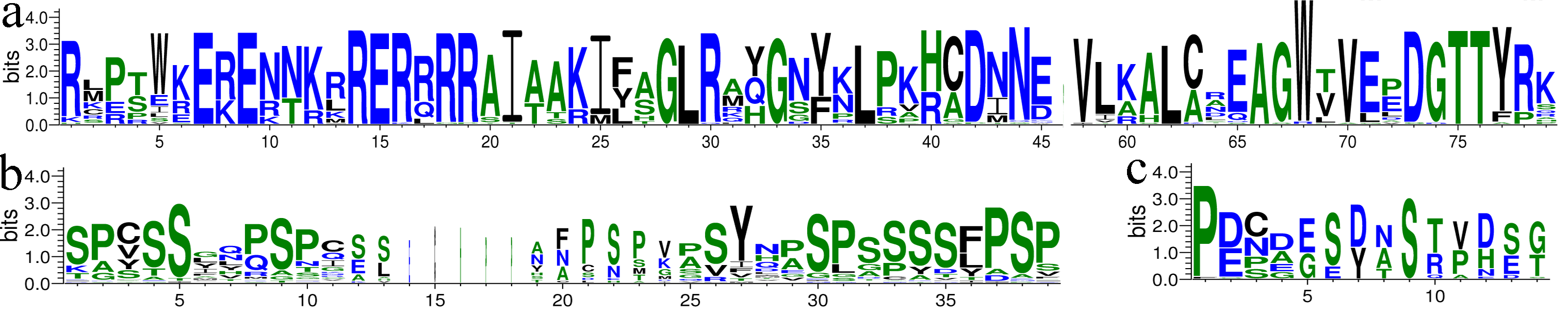

Supplement: Figure S4 — Intraspecies microsynteny of BZR gene families in P. bretschneideri and P. communis. Genomic fragments are suggested by numbers of triangles. Black triangle represented BZR genes, and same color indicated these genes in the same fragment. [file Image_4.tif]

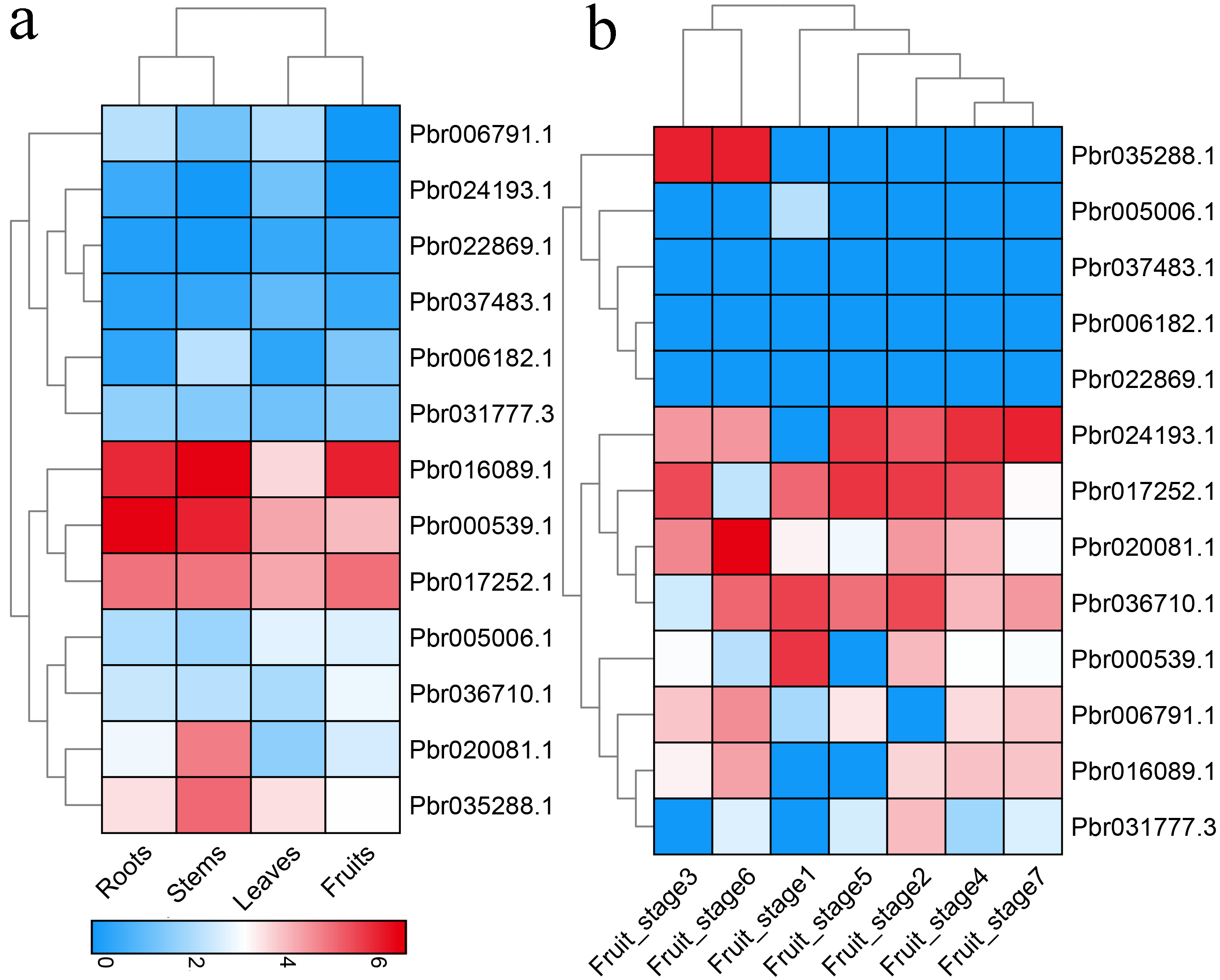

Supplement: Figure S5 — The expression of PbBZRs in different tissues (a) and fruit development stages (b). The spectrum from blue to red indicates the relative expression level from 0 to 6. Fruit_stage1, Fruit_stage2, Fruit_stage3, Fruit_stage4, Fruit_stage5, Fruit_stage6 and Fruit_stage7 indicated 15 days after flower (DAF), 30 DAF, 55 DAF, 85 DAF, 115 DAF, 130 DAF and145 DAF, respectively. [file Image_5.tif]

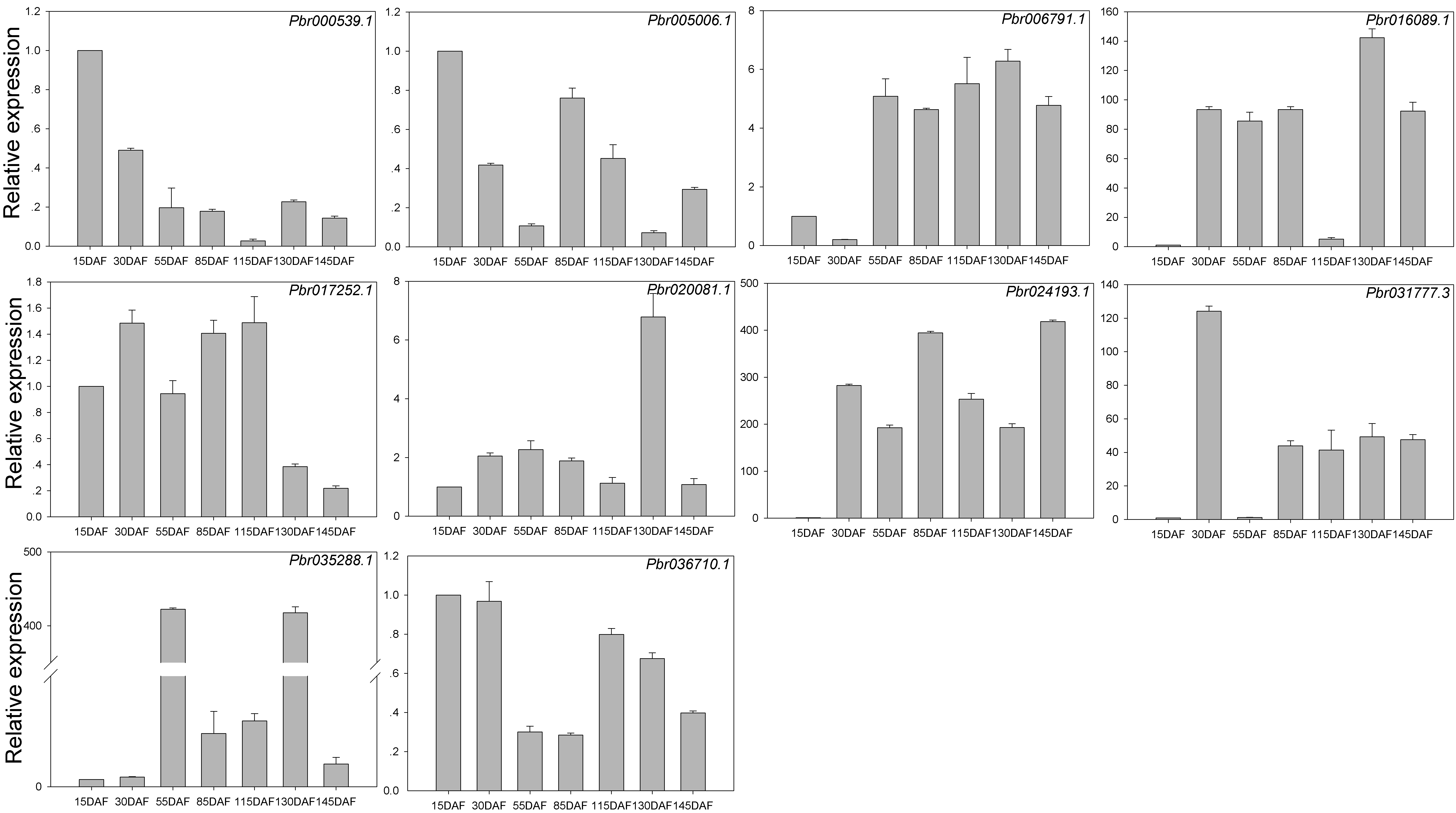

Supplement: Figure S6 — qRT-PCR verification of 10 pear genes in seven developmental stages of pear fruits, including 15 days after flower (DAF), 30DAF, 55DAF, 85DAF, 115DAF,130DAF and 145 DAF. [file Image_6.tif]

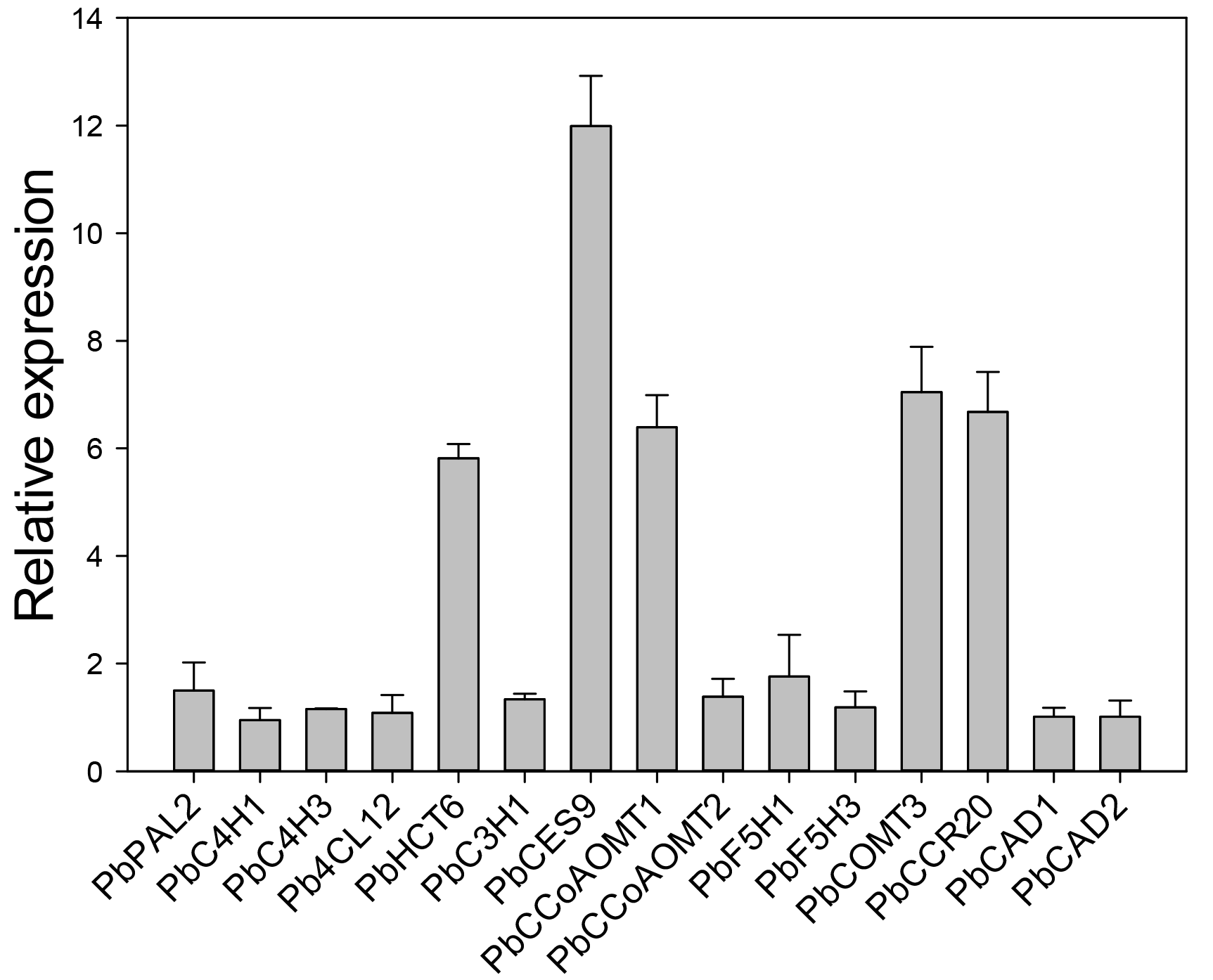

Supplement: Figure S7 — qRT-PCR assay of key genes in P. bretschneideri lignin metabolic pathway. The Y-axis indicates the relative expression levels. The expression of each gene in control fruits was set as 1. Each value is the mean ± SE of three biological replicates. [file Image_7.tif]
